# Infection risk varies within urbanized landscapes: the case of coyotes and heartworm

**DOI:** 10.1186/s13071-021-04958-1

**Published:** 2021-09-09

**Authors:** Katherine E. L. Worsley-Tonks, Stanley D. Gehrt, Chris Anchor, Luis E. Escobar, Meggan E. Craft

**Affiliations:** 1 Department of Veterinary Population Medicine, University of Minnesota, Saint Paul, MN 55108 USA; 2 School of Environment and Natural Resources, The Ohio State University, Columbus, OH 43210 USA; 3 Max McGraw Wildlife Foundation, Dundee Township, IL USA; 4 Forest Preserve District of Cook County, Hoffman Estates, IL 60120 USA; 5 Department of Fish and Wildlife Conservation, Virginia Tech, Blacksburg, VA 24060 USA; 6 Department of Ecology, Evolution, and Behavior, University of Minnesota, Saint Paul, MN USA

**Keywords:** Age, Home range, Pathogen, Urban, Vector, Wildlife

## Abstract

**Background:**

Urbanization can have profound effects on ecological interactions. For host–pathogen interactions, differences have been detected between urban and non-urban landscapes. However, host–pathogen interactions may also differ within highly heterogeneous, urbanized landscapes.

**Methods:**

We investigated differences in infection risk (i.e., probability of infection) within urbanized landscapes using the coyote (*Canis latrans*) and mosquito-borne nematode, *Dirofilaria immitis* (the causative agent for canine heartworm), as a case study. We focused on a coyote population in Chicago for which extensive behavioral and heartworm infection data has been collected between 2001 and 2016. Our objectives were to: (i) determine how onset and duration of the heartworm transmission season varied over the 16-year period and across the urban–suburban gradient; and (ii) investigate how heartworm infection risk in coyotes varied over the years, across the urban–suburban gradient, by coyote characteristics (e.g., age, sex, resident status), and coyote use of the urbanized landscape (e.g., use of urban areas, mosquito habitats).

**Results:**

While onset of the heartworm transmission season differed neither by year nor across the urban–suburban gradient, it was longer closer to the core of Chicago. Of the 315 coyotes sampled, 31.1% were infected with *D. immitis*. Older coyotes and coyotes sampled in later years (i.e., 2012–2016) were more likely to have heartworm. While coyote location in the urban–suburban gradient was not a significant predictor of infection, the proportion of urban land in coyote home ranges was. Importantly, the size and direction of this association varied by age class. For adults and pups, infection risk declined with urbanization, whereas for subadults it increased. Further, models had a higher predictive power when focusing on resident coyotes (and excluding transient coyotes). The proportion of mosquito habitat in coyote home ranges was not a significant predictor of infection.

**Conclusions:**

Our findings suggest that urbanization may affect host exposure to vectors of *D. immitis*, that risk of infection can vary within urbanized landscapes, and that urbanization–wildlife infection associations may only be detected for animals with certain characteristics (e.g., age class and resident status).

**Graphical abstract:**

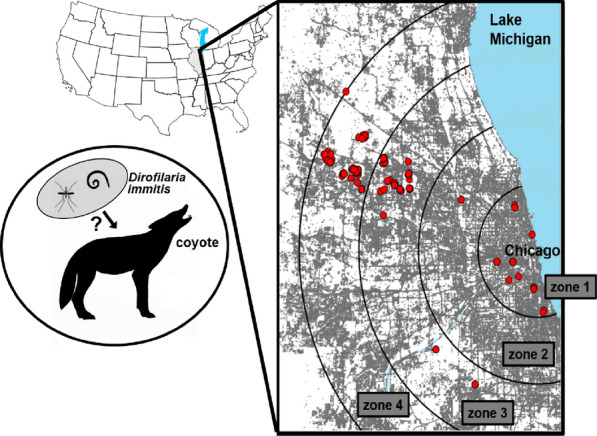

**Supplementary Information:**

The online version contains supplementary material available at 10.1186/s13071-021-04958-1.

## Background

Urbanization causes a shift in climatic conditions and landscape structure and composition [1]. Temperatures tend to increase with urbanization due to pollution and impervious surfaces [2–4]. Vegetation becomes subdivided into patches surrounded by urban and suburban blocks. This shift in environmental context can have profound effects on processes unfolding in wildlife communities [5, 6]. For example, urban-induced fragmentation of the landscape and/or warming can alter animal behavior [7, 8] and species composition and abundance [9–12], which can in turn influence ecological relationships, such as predation and competition [13, 14]. Host–pathogen interactions can also be affected by urbanization, although it may vary with context, and in many cases, depends on pathogen transmission mode (reviewed in [15–19]).

Vector-borne pathogens are prone to be affected by urbanization because of the insect vector’s dependence on appropriate habitat, warm temperatures, and competent hosts [20, 21]. Whether we should expect an increase or decrease in vector-borne diseases with urbanization is a topic of intense debate, as opposing trends have been detected [16]. For example, the prevalence of both ticks and avian malaria was found to be higher in rural common blackbirds (*Turdus merula*) than in urban ones [22]. In contrast, West Nile virus seroprevalence was greater in urban than non-urban birds (e.g., [23–25]). Importantly, in addition to contrasting large-scale outcomes across landscapes (e.g., urban vs. rural), differences in infection risk (i.e., probability of infection) can also occur within urbanized landscapes [23, 26]. For instance, differences in the number and types of mosquito habitats (e.g., wetlands, artificial containers; [27]) across neighborhoods may lead to fine-scale differences in infection outcomes [28]. While differences in infection risk within urbanized landscapes have been detected in vector species [26, 28], whether we should expect to observe similar patterns in urban host populations is less well understood.

The mosquito-borne nematode *Dirofilaria immitis* is the causative agent for canine heartworm, which is one of the most important parasitic diseases of domestic dogs in North America [29, 30]. Successful development and transmission of *D. immitis* is dependent on warm, humid conditions [31, 32], along with the presence of competent vectors and hosts [33, 34]. Warm, humid conditions are important for both the onset and duration of the heartworm transmission season and mosquito survival and reproduction. While over 60 mosquito species are susceptible to *D. immitis*, only nine act as competent vectors [35] and differ in their ability to adapt to urban settings [27]. Competent hosts include domestic dogs and wild canids, in particular coyotes (*Canis latrans*). Coyotes in rural or natural areas are considered one of the primary reservoir hosts for *D. immitis* [36–38]. *D. immitis* prevalence can be as high as 37% in coyotes sampled in some rural areas [36, 39]. Despite an increasing presence of coyotes in many urban settings [40, 41], the distribution and prevalence of *D. immitis* in urban coyote populations is relatively unknown [41].

Here, we investigated how urbanization influences coyote risk of infection with *D. immitis*. To do this, we leveraged animal behavior and infection data from a well-studied coyote population sampled between 2001 and 2016 in the northwestern portion of the Chicago metropolitan area, which included both urban and suburban regions. Historically, coyotes were rare in the Chicago area, but they increased dramatically during the 1990s and are now common throughout the metropolitan area [42]. While *D. immitis* prevalence in Chicago is unknown, the number of domestic dog cases reported by veterinary clinics in the Chicago area has increased by over four-fold in the past decade [43]. Our objectives were to (i) determine how onset and duration of the heartworm transmission season varied over the 16-year period and across the urban–suburban gradient; and (ii) investigate how infection risk in coyotes varied over the years, across the urban–suburban gradient, by coyote characteristics (e.g., age, sex), and coyote use of the urbanized landscape (e.g., use of urban areas, mosquito habitats). Because the location and size of resident coyote home ranges vary less than those of transient coyotes [42, 44], we explored coyote use of the urbanized landscape for both resident and transient coyotes and for resident coyotes only.

## Methods

### Study area

The Chicago metropolitan area, with a human population of > 9 million people, extends across six counties (i.e., Cook, DuPage, Kane, Lake, McHenry, Will) in northeastern Illinois, USA (41.88° N, 87.63° W). Chicago has a temperate climate, with mean summer and winter temperatures ranging from 26 to 33 °C and from −1 to 3 °C, respectively, and rainfall averaging ~ 845 mm per year [45]. Land cover in the region includes urban, suburban, natural areas, and agriculture. Landscapes in natural and urbanized areas include deciduous and coniferous forest, prairie, floodplain, wetland, open water, and managed green spaces (e.g., parks, greenways, golf courses).

The core of the metropolitan area, downtown Chicago, is situated on the edge of Lake Michigan. While proximity to a major water body can create a cooling effect and cause urban heat to shift westward [46], Chicago generally experiences only mild cooling that is most pronounced near the lakeshore during the summer [47] (although see [48]). This is in part due to Lake Michigan’s downwind location from the southwest winds as well as warm water temperatures in late summer [47]. Further, any cooling effect of Lake Michigan in the core of Chicago is apparently counteracted by densely populated buildings, industrial zones, and train stations [49]. Thus, Chicago’s heat island most likely occurs in the core of Chicago like traditional urban centers [1, 49], although low summer lake temperatures may push the heat island westerly.

### Heartworm transmission season

Once infected, the temperature of the mosquito dictates the development of the microfilaria to the infective third stage [50–52]. Microfilarial development occurs above a threshold temperature of 14 °C, the progress of which can be tracked through the accumulation of heartworm development units (HDUs; [53]) such that 1 HDU is equal to 1 day with an average temperature 1 °C above 14 °C [53]. Infective third-stage larvae will pass from the mosquito to the new host during blood meals only after enough HDUs have been accumulated throughout a given period [53]. The period during which infective larvae are transmitted is called the heartworm transmission season and can be constructed for any region given sufficient climatological data [50–52]. The heartworm transmission season is said to have begun when 30-day HDUs surpass 130 °C and ends when 30-day HDUs drop below 130 °C [53].

**Fig. 1:**
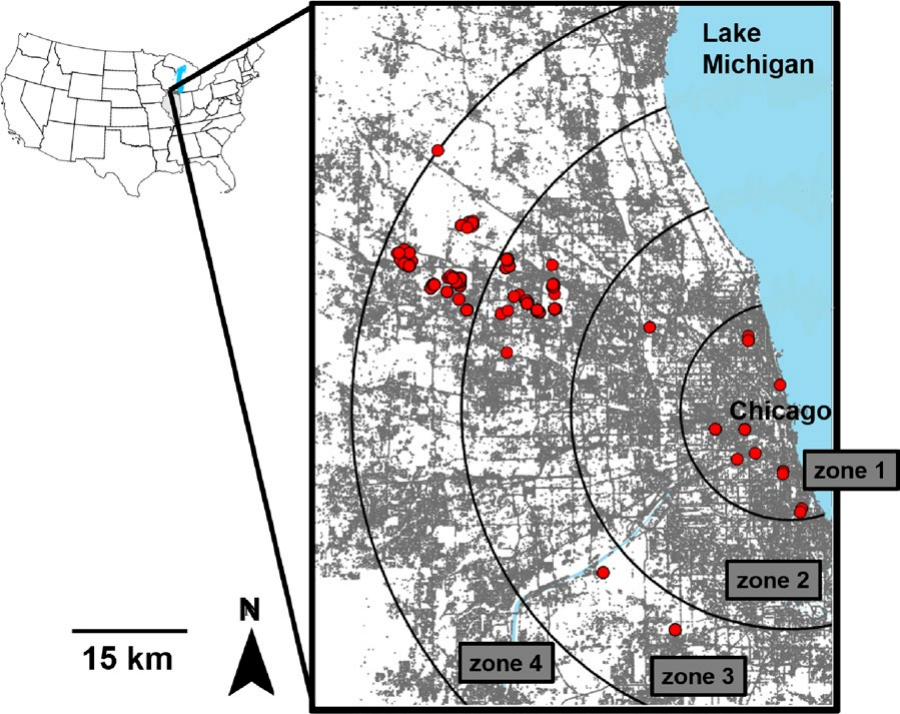
Map of the Chicago metropolitan area. Grey land represents built-up/developed land and roads (i.e., impervious surfaces from the 2011 National Land Cover Database). Red circles are coyote capture locations (*n* = 315). Zones 1–4 delineated by black circular lines were generated to explore the relationship between urbanization and the onset and duration of the heartworm transmission season. The heartworm transmission season was estimated by collecting daily temperature data from each zone (see Additional file 1: Figure S1)

To investigate the influence of urbanization on the onset and duration of the heartworm transmission season, we created four zones, each 15 km wide, to characterize a gradient from urban to suburban landscapes. Zone 1 had an average housing density of ~ 3250/km^2^, zone 2 of ~ 850/km^2^, zone 3 of ~ 530/km^2^, and zone 4 of ~ 400/km^2^ (Fig. 1) based on the 2010 SILVIS housing density data set (SILVIS Lab Spatial Analysis for Conservation and Sustainability). We obtained daily mean temperature data for the years 2000–2015 from the PRISM Climate Group (PRISM Climate Group, Oregon State University, http://prism.oregonstate.edu). Daily mean temperatures are available as spatial grids of 4-km spatial resolution which are calculated by interpolating climate data obtained from weather monitoring networks. For each zone, we used three 4-km grids that were evenly distributed across the zone (Additional file 1: Figure S1). For each 4-km grid in each year, daily HDUs were calculated by subtracting the threshold temperature of 14 °C from the daily mean temperature [53]. Thirty-day HDUs were constructed by summing each daily HDU with the daily HDUs from the previous 29 days [52, 53,54]. The duration of each heartworm transmission season was determined by the number of months between the initiation and termination of the heartworm transmission season.

### Coyote sampling

Coyotes were captured between February 2001 and December 2016 in the central and northwestern portion of the Chicago metropolitan area (Fig. 1). Most coyotes were captured in forest preserves, golf courses, small woodland parks, or in abandoned lots. Captures were performed opportunistically throughout the year, but primarily during winter and early spring. Coyotes were live-trapped with padded foothold traps and cable restraint devices [55]. With the exception of 19 coyotes, capture locations were recorded using a handheld GPS. For the 19 coyotes without specific location coordinates, we used the coordinates of the center of the park where trapping occurred. All captured individuals were transported in a metal dog carrier to a research laboratory. Each coyote was sedated while in the dog carrier with 2.5 mg/kg Telazol (Fort Dodge Animal Health, Fort Dodge, IA, USA), which was administered intramuscularly in the hind limb. After immobilization, each coyote was sexed, and aged based on reproductive condition and tooth wear [ 56, 57]. Pups were 6–12 months, subadults were between 1 and 2 years, and adults were > 2 years. Pups less than 6 months were excluded since the prepatent period for heartworm is ~ 6 months [58]. Approximately 3 ml of blood was collected from each coyote and poured into serum separator tubes. Tubes were left for ~ 30 min in an upright position and allowed to clot before being centrifuged for 15 min at 1790×*g*. Serum was extracted and stored in cryovial tubes at −80 °C. Serum was left in the freezer for periods ranging from 6 months to 5 years prior to further analyses. After blood collection, all coyotes were ear tagged and fitted with very-high-frequency radio collars (Advanced Telemetry Systems, Isanti, MN, USA). After recovering from immobilization, animals were released at the capture locations.

### Heartworm screening

Serum samples were submitted to the Veterinary Diagnostic Laboratory at the University of Illinois Urbana-Champaign for heartworm screening. Presence of heartworm antigen was assessed using a membrane-bound ELISA test (SNAP® 4Dx® Plus Test, IDEXX Laboratories Inc.) following the manufacturer’s instructions. SNAP® 4Dx® Plus Test detects proteins produced within the reproductive tract of adult female heartworms and has a sensitivity of 97.5% (95% CI 94.26–99.18) and specificity of 94.0% (95% CI 83.45–98.75) [59].

### Home range analysis and resident status classification

Relocations of all radio-collared coyotes were recorded 2–3 times a week during the day, and once a week during the night [42]. Relocations were estimated using triangulation (with program LOCATE II; Pacer, Truro, Nova Scotia, Canada) with a truck-mounted antenna, or visual sightings. Relocations were used to estimate annual home ranges. We restricted annual home range estimates to individuals with a minimum of 30 relocations during at least 6 consecutive months. All relocations recorded beyond the 12-month period post capture were excluded. We assumed that the land cover types used by each coyote 6–12 months post capture were similar to those used when exposed to vectors of *D. immitis*.

We used two nonparametric methods to obtain home range estimates for each coyote: (1) we calculated and plotted 95% minimum convex polygons (MCPs); and (2) we used the adaptive local convex hull (a-LoCoH) method [59, 61]. For a-LoCoH, we calculated 95% contours and obtained the value of the adaptive sphere of influence “*a*” by calculating the maximum distance between two points [61]. We used MCP and a-LoCoH over other home range estimators (e.g., the kernel density estimator) because MCP is most frequently used for very-high-frequency data, and a-LoCoH minimizes the extent to which home ranges cross hard boundaries (e.g., highways, rivers) [42, 60]. Since MCP can overestimate home range size [61] and a-LoCoH underestimate home range size (particularly if the sample size of locations is relatively small) [61], we explored heartworm–land cover associations using both methods. Because results were similar across methods (see “Results”), we presented MCP results in the main text and a-LoCoH results in the supplementary materials. All home range analyses were performed using the “*adehabitatHR*” package [62] in the statistical program R version 4.0.2 [63].

Home ranges were imported into ArcGIS version 10.3 [64] and linked to land cover data to estimate the proportion of each land cover type present within each coyote’s home range. We used the 2011 National Land Cover Database (https://www.mrlc.gov) (spatial resolution: 30 m) to subdivide the landscape into different land cover types. Fourteen land cover types were present in the area. We combined eleven of these into two categories: (1) “mosquito habitat” (open water, woody wetlands, and emergent herbaceous wetlands); and (2) “green spaces” (open developed space, mixed forest, evergreen forest, deciduous forest, cultivated crops, pasture/hay, grassland/herbaceous, shrub/scrub). The other three were urban land cover types (high, medium, and low developed urban land) and were examined as independent variables since we were interested in the degree of urbanization. High, medium, and low developed urban land indicates that 80–100%, 50–79%, and 20–49% of the land is impervious surface, respectively. Because all three urban variables were highly correlated with the “green spaces” variable and the “high developed urban land” variable with the “medium developed urban land” variable (*r*^2^ > 0.5), we excluded the “green spaces” and "high developed urban land” variables from statistical analyses and focused on “mosquito habitat,” “developed medium urban land,” and “developed low urban land” variables.

We used multiple characteristics to discriminate resident and transient coyotes. Residents repeatedly used an explicit territory across two or more seasons, and transients shifted use areas across seasons and had larger home ranges that overlapped multiple territories [42, 65, 66]. Further, residents were often seen traveling with other coyotes, whereas transients did not, or residents shared the same territories with reproductive pairs occupying a territory.

### Statistical analysis

We ran a linear regression to determine how the duration of the heartworm transmission season varied by year and urban zone (Table 1). The outcome variable in this model was duration of the heartworm transmission season (in months), and predictor variables were year (2001–2015) and urban zone (1–4). We also included latitude to account for any variation associated with collecting temperature data from three 4-km grids located at different latitudes within each zone (Additional file 1: Figure S1). Variation in the onset of the heartworm transmission season with year, urban zone, and latitude was not explored because there was little variation (i.e., onset of the heartworm transmission season occurred in June 97.9% of the time).

**Table 1:** Description of statistical approaches used

Analytical approach	*n*	Outcome variable	Fixed effects	Random effect(s)
Linear regression model	192	Duration of the heartworm transmission season (months)	Year (2000–2015) Urban zone (1–4) Latitude	NA
Binomial generalized linear mixed model	315^a^	Infection (yes/no)	Year (2001–2016; no heartworm data were collected in 2006 and 2007) Age class (pup (6–12 months), juvenile, adult) Sex Urban zone (1–4) Proportion of adults tested each year (as an offset)	Site Animal ID
Binomial generalized linear mixed model^b^	146	Infection (yes/no)	Year Age class Resident status (resident vs. transient)^c^ Proportion low developed urban land in home range Proportion medium developed urban land in home range Proportion mosquito habitat in home range Proportion of adults tested each year (as an offset) Age class * proportion low developed Age class * proportion medium developed Age class * proportion mosquito habitat	Site

^a^Sixteen of the coyotes were captured more than once

^b^Four models were run using this model structure and composition: (1) for residents and transients using MCP; (2) for residents only using MCP; (3) for residents and transients using a-LoCoH; and (4) for residents only using a-LoCoH

^c^Variable was included only when both resident and transient coyotes were analyzed

We ran a generalized linear model (GLM) to investigate how heartworm infection risk in coyotes varied across years (2001–2016), the urban–suburban gradient (urban zone 1–4), with coyote characteristics (e.g., age, sex), and coyote use of the urbanized landscape (i.e., mosquito habitat, medium developed urban land, and low developed urban land). However, we split the analysis into two because of differences in sample size for some of the fixed effects. One analysis included all coyotes tested for heartworm (*n* = 315 coyotes with 16 tested more than once) and the other included coyotes for which enough relocation data were obtained to estimate annual home ranges (*n* = 146 coyotes, a subset of the 315 coyotes). The outcome variable was heartworm infection (yes/no), thus we ran binomial GLMs with logit link functions using the “*lme4*” R package [67]. Additionally, because we expected infection to increase with age [38, 39, 68], we also included the proportion of adults tested each year as an offset in both analyses (Table 1).

In the analysis that included 315 coyotes, urban zone, age class, and sex were included as categorical fixed effects. Year was included as a continuous fixed effect, and nonlinear relationships with heartworm infection were examined using basis splines (Table 1) using the “s*plines*” R package. Since 16 coyotes were tested more than once (Additional file 1: Table S1), we include “animal ID” as a random intercept and ran a generalized linear mixed model (GLMM) instead of a GLM using the “*lme4*” package. Further, we grouped observations into a “site” random intercept to account for any significant spatial autocorrelation in model residuals (Moran’s *I* statistic after including site as a random effect: *z* = 0.52, *P* = 0.3). Coyotes were included in the site that was closest to their capture location except if there were man-made barriers (e.g., highways).

In the analysis that included 146 coyotes, we ran four models: (1) for residents and transients using MCP; (2) for residents only using MCP; (3) for residents and transients using a-LoCoH; and (4) for residents only using a-LoCoH. Fixed effects included the proportion of mosquito habitat, medium developed urban land, and low developed urban land in coyote home ranges. Age class and year were included as fixed effects because they were significant predictors in the analysis with 315 coyotes (see “Results”). Resident status was also included in models that examined both residents and transients. Since we expected age class to be an important predictor of infection, we also evaluated whether the association between infection and the proportion of mosquito habitat, medium developed urban land, and low developed urban land in coyote home ranges varied by age class by including interactions between these variables. We included site as a random intercept to account for any significant spatial autocorrelation (Moran’s *I* statistic after including site as a random effect for residents and transients using MCP: *z* = 0.08, *P* = 0.47; for residents only using MCP: *z* = −0.24, *P* = 0.59; for residents and transients using a-LoCoH: *z* = −0.02, *P* = 0.51; for residents only using a-LoCoH: *z* = −0.36, *P* = 0.64). Animal ID was not included as a random intercept because none of the coyotes in this second analysis were resampled more than once.

For all models, the most parsimonious model was identified using an information theory approach, comparing models with different variable combinations, and used the Akaike information criterion corrected for small sample size (AICc) to rank models [69, 70] using the “*MuMIn*” R package [71]. If at least one model was within 2 ΔAICc values of the top-ranking model, model averaging was used to obtain mean effect sizes and 95% confidence intervals [69]. All continuous predictors were centered and standardized to facilitate interpretation of main effects and to perform model averaging [72]. Multicollinearity among continuous predictors was assessed using the variance inflation factor [73]. Scaled residuals of each model were examined for uniformity using the “*DHARMa*” package [74]. Model fit was assessed by calculating the marginal and conditional coefficients of determination (*r*_m_^2^ and *r*_c_^2^, respectively) [75]. *r*_m_^2^ is the variance explained by the fixed effects, and *r*_c_^2^ the variance explained by the fixed and random effects [75].

## Results

### Onset and duration of the heartworm transmission season

**Fig. 2:**
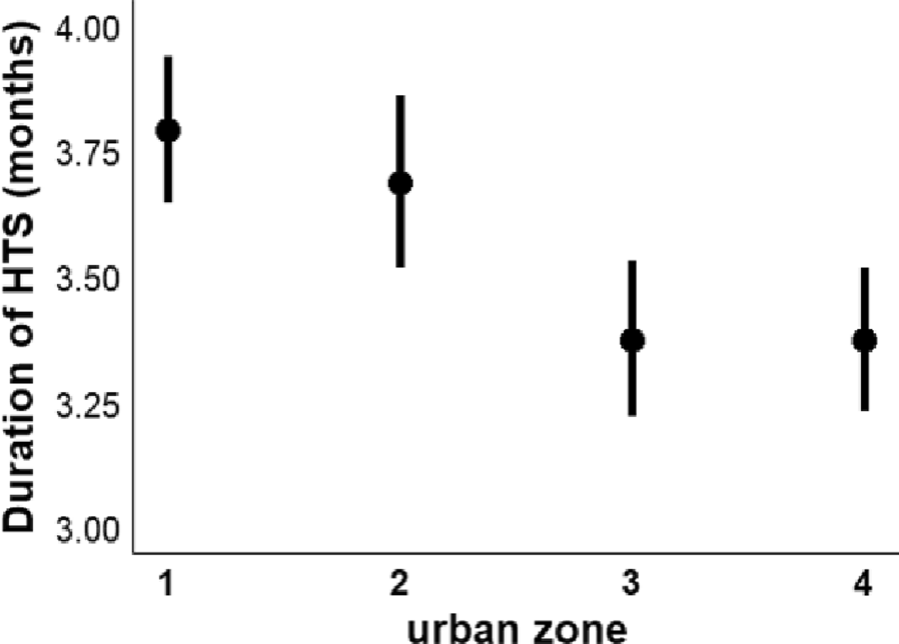
Duration of the heartworm transmission season (in months) for each urban zone. Zone 1 is closest to the core of Chicago, and zone 4 is furthest away (Fig. 1, Additional file 1: Figure S1). Whiskers are 95% confidence intervals

Across all urban zones (1–4) and years (2000–2015), the heartworm transmission season most often began in June (97.9% of the time) and lasted for a period of 2–5 months (mean of 3.56 months). For the duration of the heartworm transmission season, urban zone appeared in all three of the top-ranking models, and year and latitude in one (Additional file 1: Table S2). Model averaging of the top three models showed that urban zone was a significant predictor of infection (Table 2), while year and latitude were not (Table 2). The heartworm transmission season was significantly longer in zone 1 compared to zone 3 and 4 (Fig. 2). Pairwise comparisons revealed that the same was true for zone 2 compared to zone 3 and 4 (zone 2 vs. zone 3: *z* = 2.88, *P* = 0.02; zone 2 vs. zone 4: *z* = 2.89, *P* = 0.02).

**Table. 2:** Model averaging results from the linear regression model of the duration of the heartworm transmission season (*n* = 192)

Predictors	Estimate	SE	*z*	Pr(> |*z*|)	95% CI
(Intercept)	3.79	0.08	48.79	< 0.001	3.63 to 3.93
Urban zone 2	−0.1	0.11	0.9	0.37	−0.31 to 0.12
Urban zone 3	−0.41	0.11	3.74	< 0.001	−0.63 to −0.2
Urban zone 4	−0.41	0.11	3.74	< 0.001	−0.63 to −0.2
Latitude	−0.04	0.04	0.94	0.35	−0.12 to 0.04
Year	0.03	0.04	0.84	0.4	−0.04 to 0.11

Predictors were obtained from the top-ranking models (ΔAICc < 2; Additional file 1: Table S2)

For urban zone, zone 1 is the reference level.  Significant terms are those for which 95% confidence intervals [CI] do not overlap with 1 and P < 0.05.

### Urbanization and coyote infection risk

**Fig. 3:**
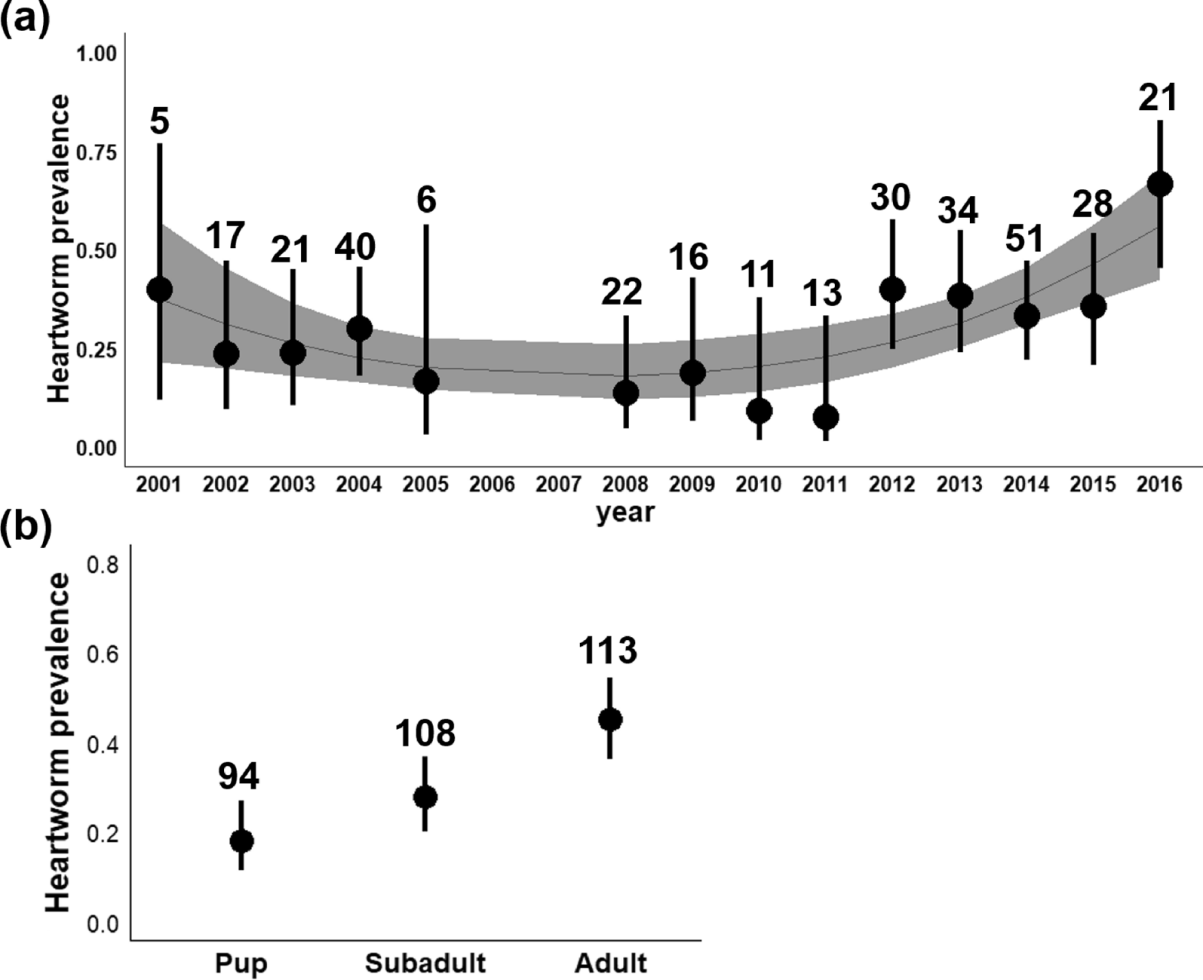
Relationship between heartworm prevalence and **a** year and **b** age class. Whiskers are 95% confidence intervals. In panel **a**, the line denotes the mean infection risk by year based on a quadratic model. The shaded area denotes the 95% confidence interval, and numbers are sample sizes. No heartworm data were collected in 2006 and 2007.

Three hundred and fifteen coyotes were captured and tested for heartworm between 2001 and 2016 (16 were captured more than once; Additional file 1: Table S1). The number of animals captured and tested each year ranged from 5 in 2001 to 51 in 2014 (mean = 22.5 per year). Heartworm tests were performed on 94 pups (52 females and 42 males), 108 subadults (53 females and 55 males), and 113 adults (38 females and 75 males). Ninety-eight coyotes were positive for heartworm (31.1%). Prevalence ranged from 7.7% in 2011 (*n* = 13) to 66.7% in 2016 (*n* = 21) (Fig. 3a).

When examining infection risk for all captured coyotes (*n* = 315), the best fit model contained only age class and year (Additional file 1: Table S3). Urban zone and sex were not important predictors of infection because they did not appear in the top-ranking model (Additional file 1: Table S3). A quadratic relation better explained the relationship between heartworm infection and year than a linear relationship (Table 3). Infection risk was lowest in 2008–2011 and increased in 2012–2016 (Fig. 3a). For age class, adults had a higher risk of infection than pups and subadults (Table 3; Fig. 3b).

**Table. 3:** Relationship between heartworm infection and coyote age class and year (*n* = 315)

Predictors	Estimate	SE	*z*	Pr(> |*z*|)	OR	95% CI
(Intercept)	−0.52	0.56	−0.93	0.35	0.6	(0.2–1.79)
Age class (subadult)	−1.005	0.35	−2.87	0.004	0.37	(0.18–0.73)
Age class (pup)	−1.74	0.41	−4.2	< 0.001	0.18	(0.08–0.4)
Year	−2.14	1.12	−1.9	0.06	0.12	(0.01–1.07)
Year (quadratic)	1.44	0.52	2.75	0.006	4.21	(1.5–11.9)

Predictors were obtained from the best fit GLMM (Additional file 1: Table S3)

For age class, adult is the reference level. Significant terms are those for which  95% confidence intervals [CI] do not overlap with 1 and *P* < 0.05. *SE* is the standard error, Pr(> |z|) the *P*-value associated with the *z* statistic, and *OR* the odds ratio

**Fig. 4:**
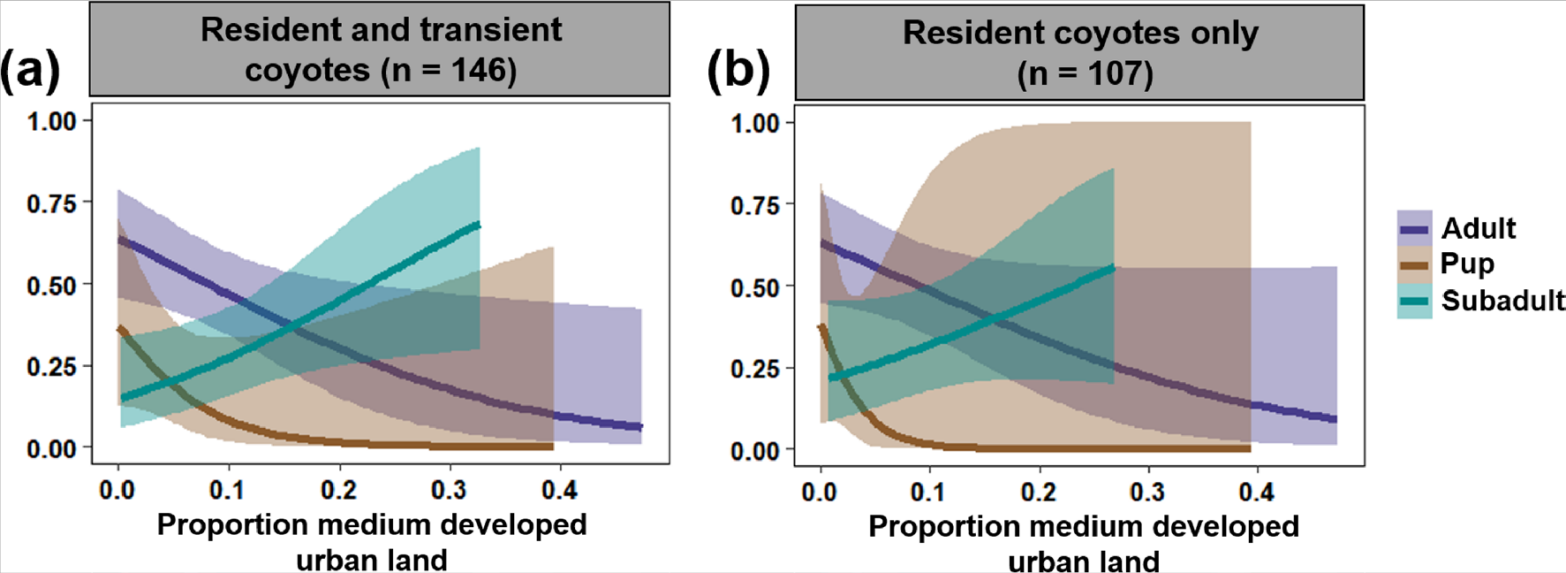
Relationship between infection risk and the proportion of medium developed urban land in coyote home ranges by age class. Panel **a** includes both resident and transient coyotes (*n* = 146), and panel **b** resident coyotes only (*n* = 107). Lines are mean estimates of infection risk (i.e., probability of infection) by proportion of medium developed urban land in coyote home ranges. The shaded bands are 95% confidence intervals. Coyote home ranges were estimated using 95% minimum convex polygons (95% MCPs). For results using a-LoCoH, see Additional file 1: Figure S2

Of the 315 coyotes tested for heartworm, 146 had enough relocations to estimate annual home ranges (mean number of relocations per animal = 163, range = 45–585). The 146 individuals comprised 37 pups (21 females and 16 males), 46 subadults (23 females and 23 males), and 63 adults (25 females and 38 males). In terms of resident status, this amounted to 107 residents and 39 transients. The years with the lowest number of coyotes tracked were 2005 and 2010 (*n* = 3), and the years with the highest number of coyotes tracked were 2012 and 2013 (*n* = 22) and 2014 (*n* = 23). Association between heartworm infection and age class, year, resident status, and coyote use of the urbanized landscape tended to be similar across all four models (i.e., resident and transient coyotes using MCP, resident coyotes only using MCP, resident and transient coyotes using a-LoCoH, and resident coyotes only using a-LoCoH). However, models with the greatest predictive power used MCP instead of a-LoCoH and focused on resident coyotes only. For top-ranking models using MCP (ΔAICc < 2), the largest *r*^2^ value was 0.68 for the resident coyotes only analysis and 0.49 for the resident and transient coyote analysis. For top-ranking models using a-LoCoH, the largest *r*^2^ value was 0.47 for the resident coyotes-only analysis and 0.5 for the resident and transient coyote analysis (Additional file 1: Table S3). Results using MCP are summarized in Table 4, Fig. 4, and Additional file 1: Table S4, and results using a-LoCoH are summarized in Additional file 1: Tables S4, S5, and Figure S2.

**Table 4:** Model averaging results from binomial generalized linear mixed models of heartworm infection risk in coyotes (*n* = 146)

Model	Predictor	Estimate	SE	*z*	Pr(> |*z*|)	Mean OR	95% CI
Resident and transient coyotes (*n* = 146)	(Intercept)	−0.59	0.45	1.33	0.18	0.55	(0.23–1.33)
	Age class (subadult)	−0.75	0.46	1.64	0.1	0.47	(0.19–1.16)
	Age class (pup)	−2.37	1.02	2.33	0.02	0.09	(0.01–0.69)
	Prop. low developed in home range	−0.22	0.27	0.82	0.41	0.8	(0.48–1.36)
	Prop. medium developed in home range	−0.63	0.33	1.9	0.06	0.53	(0.28–1.02)
	Prop. mosquito habitat in home range	0.33	0.23	1.46	0.15	1.4	(0.89–2.19)
	Year	−1.74	1.53	1.14	0.26	0.18	(0.01–3.54)
	Year (quadratic)	0.75	0.65	1.15	0.25	2.12	(0.59–7.64)
	Age class (subadult) * proportion medium developed	1.48	0.48	3.06	0.002	4.39	(1.7–11.3)
	Age class (pup) * proportion medium developed	−1.26	1.33	0.95	0.34	0.28	(0.02–3.84)
Resident coyotes only (*n* = 107)	(Intercept)	−0.49	0.3	1.62	0.1	0.61	(0.35–1.08)
	Age class (subadult)	−0.75	0.5	1.5	0.13	0.48	(0.18–1.27)
	Age class (pup)	−3.49	2.66	1.31	0.19	0.02	(0.00–4.48)
	Proportion medium developed in home range	−0.56	0.34	1.64	0.1	0.57	(0.29–1.12)
	Proportion mosquito habitat in home range	0.28	0.25	1.14	0.26	1.32	(0.82–2.14)
	Age class (subadult) * proportion medium developed	1.16	0.56	2.07	0.04	3.19	(1.06–9.53)
	Age class (pup) * proportion medium developed	−3.36	3.83	0.88	0.38	0.03	(0.00–62.73)

Predictors were obtained from the top-ranking models (ΔAICc < 2; Additional file 1: Table S4). Coyote home ranges were estimated by calculating and plotting 95% minimum convex polygons (MCPs). Results using 95% adaptive local convex hulls (a-LoCoH) are summarized in Additional file 1: Table S4 and S5)

*SE* is the standard error, Pr(> |*z*|) the *P*-value associated with the *z* statistic, and mean *OR* the mean odds ratio

Age class, proportion of medium developed urban land in coyote home ranges, and the interaction between these two predictors appeared in all top-ranking models (Additional file 1: Table S4, except for resident coyotes only using MCP in which two out of three models had the two predictors and interaction) and thus were the most important predictors of heartworm infection. Further, for the resident and transient models, proportion of mosquito habitat and year were second most important, followed by proportion of low developed urban land in coyote home ranges and resident status (Additional file 1: Table S4). For the resident only models, year appeared in none of the top-ranking models (Additional file 1: Table S4). The interaction between age class and proportion of low developed urban land, and mosquito habitat in coyote home ranges were the least important as they did not appear in any of the top-ranking models (Additional file 1: Table S4).

Model averaging revealed that when resident and transient coyotes were examined, pups had a lower infection risk than adults (Table 4 and Additional file 1: Table S5). Further, infection risk tended to decline as the proportion of medium developed urban land in coyote home ranges increased. However, this association varied by age class, where infection risk declined as the proportion of medium developed urban land in home ranges increased for adults but increased for subadults (Table 4, Fig. 4, and Additional file 1: Table S5 and Fig. S2). Further, changing the model reference level to pups revealed that, for the models that included both resident and transient coyotes, the relationship between infection and proportion of medium developed urban land in home ranges was significantly different between pups and subadults (*P* < 0.05 for all four model types), where pups, like adults, had a lower risk of infection with more medium developed urban land in their home ranges (Fig. 4 and Additional file 1: Table S2). No significant difference was detected between pups and subadults when focusing on resident coyotes only.

## Discussion

Urbanization can have contrasting effects on host–pathogen interactions [15, 18 19]. Here, we found that urbanization influenced the duration of the heartworm transmission season and infection risk in coyotes. The heartworm transmission season was longer closer to the core of Chicago. Heartworm prevalence in coyotes increased during the study period and with coyote age. Further, the proportion of medium developed urban land in coyote home ranges was an important predictor of infection, but direction and size of the effect varied by age class and models had a higher predictive power when examining resident coyotes only. For adults and pups, infection risk declined with urbanization, whereas for subadults, it increased.

The Chicago coyote population had a heartworm prevalence of 31.1%. Previous studies performed in Madison, Wisconsin and Tucson, Arizona have found heartworm prevalence in urban coyotes to be 35.7% (*n* = 14) [76] and 0% (*n* = 22) [77], respectively. Importantly, we found that heartworm prevalence fluctuated yearly, with prevalence being as low as 8% in some years and as high as 67% in others, suggesting that there can be notable differences in prevalence across years. Further, heartworm prevalence tended to increase over the 16-year period, a finding that is in line with national trends observed in domestic dogs suggesting that heartworm prevalence is increasing over time across the USA [30]. For northern US states like Illinois, this increase may be associated with an increase in the number and density of mosquito vectors [78, 79], possibly due to the combined effect of shifting climate conditions and few mosquito abatement programs [30]. For Chicago, an increase in coyote numbers over the years [42] could also be an important factor.

The proportion of coyotes sampled closer to the core of Chicago also increased over the years, which may have contributed to an increase in heartworm prevalence over time. Twenty-six coyotes were sampled in the urban zone 1, of which 24 (92%) were sampled in 2013–2016. While urban zone was not a significant predictor of infection, coyote proximity to the core of Chicago may play a role because the heartworm transmission season tended to be longer closer to the core of Chicago. Additionally, heartworm prevalence in rural coyotes in Illinois is 16% [38], suggesting that urban coyotes might be at a higher risk of infection than non-urban coyotes. Further, mosquitoes sampled in urban areas can have a higher heartworm prevalence than rural mosquitoes [80]. This could be because one of the main vectors of *D. immitis*, *Aedes albopictus*, tends to thrive in urbanized landscapes owing to warmer conditions and the presence of natural and artificial water bodies and containers [35, 80, 81]. A nonsignificant effect of urban zone may be due to a smaller sample size closer to the core Chicago (i.e., only 29 coyotes were tested in zone 1 and 2 combined: 26 in zone 1 and three in zone 2).

Another potential reason for not detecting a significant effect of urban zone on coyote infection could be that measuring proximity of coyotes to the urban core simplifies or underestimates complex patterns occurring within urban patches. For example, mosquito abundance and richness, as well as infection, can vary across distances as small as neighborhoods [26], and the degree of landscape heterogeneity can influence mosquito diversity [82, 10]. Exploring land cover composition of coyote home ranges provided greater insight for associations with infection risk than proximity to the core of Chicago. The proportion of urban land in coyote home ranges was an important predictor of infection, but only when quantified as medium developed urban land and not low developed urban land. Since impervious surfaces account for 50–79% of total land cover for medium developed urban land and 20–49% for low developed urban land, impervious surfaces and built-up land may explain the observed association.

It was surprising that the proportion of mosquito habitats in coyote home ranges was a nonsignificant predictor of infection risk. In the case of domestic dogs, proximity to mosquito-bearing waters can be an important predictor of infection with *D. immitis* [83]. One reason for not detecting an association in our system could be that there were other unaccounted for water bodies (e.g., man-made or temporary water bodies such as artificial containers, puddles, tires, trash cans) [84, 85]. Exploring whether the presence of man-made and temporary water bodies versus permanent/vegetated water bodies in coyote home ranges influences heartworm infection would be an important next step to take.

It is noteworthy that the relationship between infection risk and the proportion of medium developed urban land in coyote home ranges varied both in directionality and size by age class. The negative association detected for adults and pups is likely associated with built-up areas having lower mosquito abundance and mosquito species richness than green spaces (e.g., parks, forest preserves; [86, 87]). There are nine competent vectors of *D. immitis* [35], most of which breed in wetland and woodland areas [88]. For example, *Ae. vexans*, a floodwater mosquito, is most frequently found in riparian zones, roadside ditches, and wetlands [35, 89], and is therefore perhaps more commonly found in urban green spaces. Adults and pups may also be at a lower risk of infection with more medium developed urban land in their home range because there is perhaps more mosquito control than in green spaces. The small effect size detected for pups is probably because pups had a lower prevalence than adults. Indeed, heartworm infection risk tended to increase with age, a pattern that is consistent with previous work [38, 39, 68]. The positive association detected for subadults could be because most subadults are transitioning between their natal and new territories. During this dispersal period, subadults may be exposed to a broader range of microhabitats than adults and pups, and thus could have more opportunities to encounter environments with more mosquitoes. An important next step that would help disentangle the importance of these various potential explanations would be to explore whether there is a relationship between infection risk and habitat use within home ranges.

The fact that the directionality of the infection–urbanization association by age class remained the same regardless of the home range estimator used (MCP vs. a-LoCoH) highlights the strength of these associations. However, it is interesting that model predictive power increased when focusing on resident coyotes and excluding transients. Because transients tended to have larger and more complex home ranges than residents, we suspect that the infection–urbanization association might differ, or might be less apparent, if a larger number of transients were included in the analysis (and/or that transients were examined separately). More research is needed to determine whether transient coyotes likely would have the same infection–urbanization association as resident adult coyotes or as subadult coyotes.

We cannot infer that adult coyotes, particularly transients, were likely infected in the areas where they were sampled; however, we can for pups, as coyotes only tend to leave their natal territories as subadults [90]. The fact that the direction of the infection–urbanization association was the same in pups and adults suggests that coyotes from these two age classes may have been infected in the area (or similar environment) where they were sampled. This lends support for the notion that urban wildlife reflect their local environment [25, 91] and highlights the need to carefully consider which types of individuals should be examined to effectively capture any associations with local environments (e.g., pups and resident adults in our case study).

While this study provides insight on how urbanization might influence wildlife infection risk, there were a number of limitations. Firstly, coyote sampling varied across years and urban zones, which limited result interpretations in some cases. That said, to the best of our knowledge, this is one of the first + 15-year urban wildlife disease investigations and provides unique evidence that wildlife disease risk can vary over time in urbanized settings. Another broader limitation worth noting was the inability to account for the time lag between human-derived changes to the landscape, and vector and wildlife response to this change [16, 33]. This could be especially important for urban and suburban land covers, which tend to increase over time. Future work should focus on developing approaches that can approximate time lags between land-use change and host and pathogen response to such changes [16]. Another important limitation of this study was not accounting for socioeconomic factors. Recent studies suggest that a number of socioeconomic factors can influence the distribution of wildlife diseases in urbanized areas (e.g., household income) [92, 93]. Future vector-borne and wildlife disease studies should quantify socioeconomic factors alongside of structural and abiotic components when exploring effects of urbanization on infection risk [94].

## Conclusions

Measuring the effects of urbanization on host–pathogen interactions is becoming an important area of research [15, 19], particularly as urbanized areas continue to expand [95]. Recent work has found differences in disease risk between urban and non-urban wildlife populations (reviewed in [18]). Here, we found that coyote infection with the vector-borne pathogen *D. immitis* can vary within urban and suburban areas, and that effects may only be detected for certain age classes, and when using certain metrics of urbanization. While we were not able to make comparisons with rural or wildland coyotes, the fact that we detected differences in infection risk among coyotes residing in different urban and suburban areas highlights the complex way by which vector-borne diseases are transmitted in urbanized landscapes.

## Supplementary Information

**Additional file 1: Figure S1.** Map of the Chicago metropolitan area. Black circular lines delineate the four urban zones. Red squares are grids where temperature data were gathered. Temperature data were obtained from the PRISM Climate Group (PRISM Climate Group, Oregon State University, http://prism.oregonstate.edu). **Figure S2.** Relationship between heartworm infection and the proportion of medium developed urban land in coyote home ranges by age class. Panel **a** includes both resident and transient coyotes (*n* = 146), and panel **b** resident coyotes only (*n* = 107). The lines are mean estimates of the probability of heartworm infection by proportion urban land in coyote home ranges. The shaded bands are 95% confidence intervals. Coyote home ranges were estimated using 95% adaptive local convex hulls (95% a-LoCoH). **Table S1.** Number of recaptured coyotes and years of captures (*n* = 16). Coyotes were grouped based on whether they tested positive or negative on the first and second occasion. **Table S2.** Linear regression models predicting the duration of the heartworm transmission season (*n* = 192). Models are ranked based on ΔAICc. **Table S3.** Generalized linear mixed models (GLMMs) predicting heartworm infection (*n* = 315). GLMMs are ranked based on ΔAICc. **Table S4.** Top twenty generalized linear mixed models predicting heartworm infection. Models within ΔAICc < 2 from the best fit model were included in model averaging. **Table S5.** Model averaging results from binomial generalized linear mixed models of the probability of heartworm infection in coyotes (*n* = 146) using the adaptive local convex hull (a-LoCoH). Predictors were obtained from the top-ranking models (ΔAICc < 2; Table S4).

## Data Availability

Data are available on figshare (10.6084/m9.figshare.15169146.v1).

## Abbreviations

HDU: Heartworm development unit
MCP: Minimum convex polygon
a-LoCoH: Adaptive local convex hull
GLM: Generalized linear model
GLMM: Generalized linear mixed model
AICc: Akaike information criterion corrected for small sample size
