# Supplementary material for: Infection risk varies within urbanized landscapes: the case of coyotes and heartworm
Source: Parasit Vectors. 2021 Sep 9;14:464. doi: 10.1186/s13071-021-04958-1 (PMC8427890; doi:10.1186/s13071-021-04958-1)
Supplement: Supplementary file 1 — Additional file 1: Figure S1. Map of the Chicago metropolitan area. Black circular lines delineate the four urban zones. Red squares are grids where temperature data were gathered. Temperature data were obtained from the PRISM Climate Group (PRISM Climate Group, Oregon State University, http://prism.oregonstate.edu). Figure S2. Relationship between heartworm infection and the proportion of medium developed urban land in coyote home ranges by age class. Panel a includes both resident and transient coyotes (n = 146), and panel b resident coyotes only (n = 107). The lines are mean estimates of the probability of heartworm infection by proportion urban land in coyote home ranges. The shaded bands are 95% confidence intervals. Coyote home ranges were estimated using 95% adaptive local convex hulls (95% a-LoCoH). Table S1. Number of recaptured coyotes and years of captures (n = 16). Coyotes were grouped based on whether they tested positive or negative on the first and second occasion. Table S2. Linear regression models predicting the duration of the heartworm transmission season (n = 192). Models are ranked based on ΔAICc. Table S3. Generalized linear mixed models (GLMMs) predicting heartworm infection (n = 315). GLMMs are ranked based on ΔAICc. Table S4. Top twenty generalized linear mixed models predicting heartworm infection. Models within ΔAICc < 2 from the best fit model were included in model averaging. Table S5. Model averaging results from binomial generalized linear mixed models of the probability of heartworm infection in coyotes (n = 146) using the adaptive local convex hull (a-LoCoH). Predictors were obtained from the top-ranking models (ΔAICc < 2; Table S4). [file 13071_2021_4958_MOESM1_ESM.docx]

**Infection risk varies within urbanized landscapes – the case of coyotes and heartworm**

Katherine E. L. Worsley-Tonks, Stanley D. Gehrt, Chris Anchor, Luis E. Escobar, and Meggan E. Craft


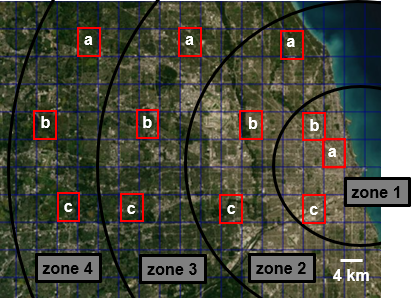


**Additional file 1: Figure S1.** Map of the Chicago metropolitan area. Black circular lines delineate the four urban zones. Red squares are grids where temperature data were gathered. Temperature data were obtained from the PRISM Climate Group (PRISM Climate Group, Oregon State University, <http://prism.oregonstate.edu>).


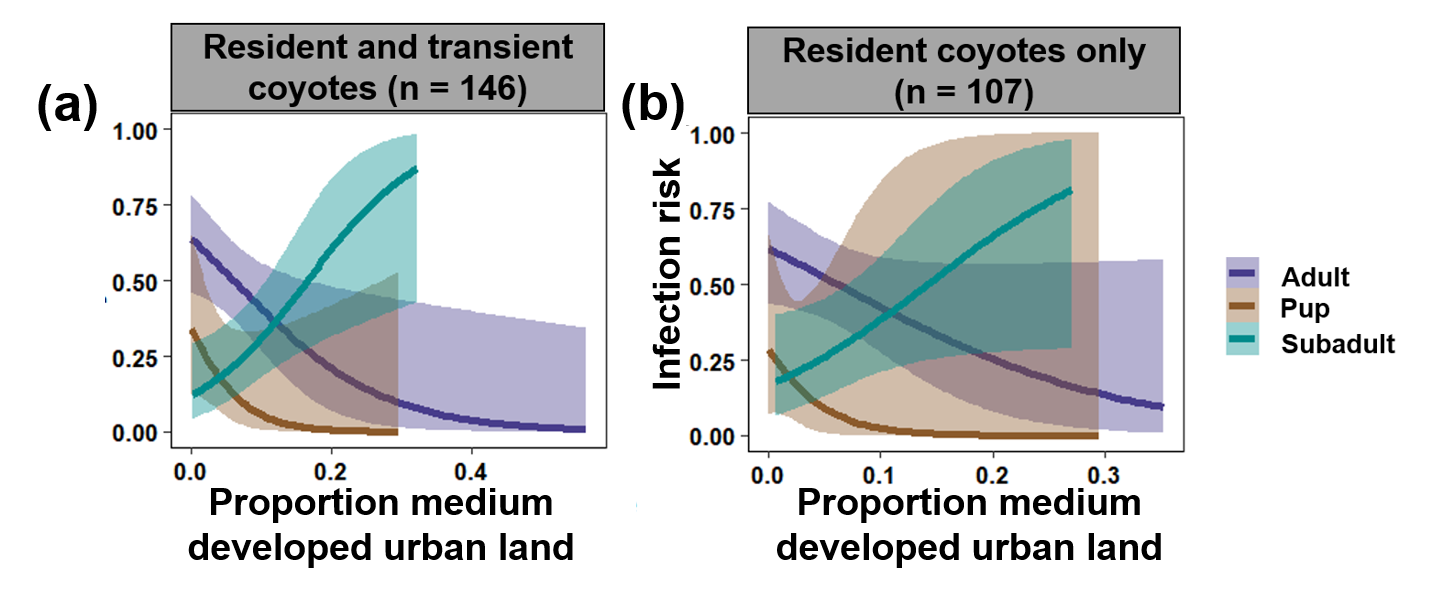


**Additional file 1: Figure S2.** Relationship between heartworm infection and the proportion of medium developed urban land in coyote home ranges by age class. Panel **a** includes both resident and transient coyotes (*n* = 146) and panel **b** resident coyotes only (*n* = 107). The line is the mean estimate of the probability of heartworm infection by proportion urban land in coyote home ranges. The shaded bands are 95% confidence intervals. Coyote home ranges were estimated using 95% adaptive local convex hulls (95% a-LoCoH)

**Additional file 1: Table S1.** Number of recaptured coyotes and years of captures (*n* = 16). Coyotes were grouped based on whether they tested positive or negative on the first and second occasion

| Category | Number of coyotes | Years of captures |
| --- | --- | --- |
| Negative to negative | 7 | 2004 and 2010 2004 and 2009 2004 and 2004 2009 and 2010  2008 and 2008 2012 and 2014 2014 and 2014 |
| Negative to positive | 4 | 2004 and 2013 2011 and 2013 2014 and 2016 2008 and 2012 |
| Positive to positive | 5 | 2003 and 2004 2004 and 2013 2013 and 2014 2014 and 2014 2016 and 2016 |
| Positive to negative | 0 | NA |

**Additional file 1: Table S2.** Linear regression models predicting the duration of the heartworm transmission season (*n* = 192). Models are ranked based on ΔAICc

| Model rank | Predictors | *k* | ΔAICc | *w_i_* | *r^2^* |
| --- | --- | --- | --- | --- | --- |
| 1 | urban zone | 5 | 0.00 | 0.43 | 0.11 |
| 2 | urban zone + latitude | 6 | 1.22 | 0.24 | 0.11 |
| 3 | urban zone + year | 6 | 1.41 | 0.22 | 0.02 |
| 4 | urban zone + latitude + year | 7 | 2.64 | 0.12 | 0.12 |
| 5 | latitude | 3 | 15.63 | 0.00 | 0.02 |
| 6 | intercept | 2 | 16.52 | 0.00 | 0.00 |
| 7 | latitude + year | 4 | 17.06 | 0.00 | 0.02 |
| 8 | year | 33 | 17.93 | 0.00 | 0.003 |

*k* is the number of coefficients, *w_i_* is the renormalized Akaike weights, and *r^2^* is the squared correlation coefficient.

**Additional file 1: Table S3.** Generalized linear mixed models (GLMMs) predicting heartworm infection (*n* = 315). GLMMs are ranked based on ΔAICc

| Model | Predictors included in each model | *k* | ΔAICc | *w_i_* | *r^2^_m_* | *r^2^_c_* |
| --- | --- | --- | --- | --- | --- | --- |
| 1 | age class + year | 7 | 0.00 | 0.65 | 0.18 | 0.32 |
| 2 | age class + year + sex | 8 | 2.1 | 0.23 | 0.18 | 0.32 |
| 3 | age class + year + urban zone | 10 | 3.89 | 0.09 | 0.19 | 0.31 |
| 4 | age class + year + sex + urban zone | 11 | 6.03 | 0.03 | 0.19 | 0.31 |
| 5 | age class | 5 | 15.2 | 0.00 | 0.09 | 0.27 |
| 6 | age class + sex | 6 | 17.14 | 0.00 | 0.09 | 0.27 |
| 7 | year | 5 | 20.24 | 0.00 | 0.08 | 0.21 |
| 8 | age class + urban zone | 8 | 20.94 | 0.00 | 0.09 | 0.27 |
| 9 | year + sex | 6 | 21.54 | 0.00 | 0.08 | 0.21 |
| 10 | age class + urban zone + sex | 9 | 22.89 | 0.00 | 0.1 | 0.26 |
| 11 | year + urban zone | 8 | 25.00 | 0.00 | 0.08 | 0.21 |
| 12 | year + urban zone + sex | 9 | 26.08 | 0.00 | 0.09 | 0.21 |
| 13 | intercept | 3 | 31.67 | 0.00 | 0 | 0.15 |
| 14 | sex | 4 | 32.23 | 0.00 | 0.007 | 0.15 |
| 15 | urban zone | 6 | 37.56 | 0.00 | 0.004 | 0.15 |
| 16 | urban zone + sex | 7 | 38.07 | 0.00 | 0.01 | 0.16 |

*k* is the number of coefficients, *w_i_* is the renormalized Akaike weights, and *r^2^_m_* and *r^2^_c_* are the marginal and conditional *r^2^* statistics, respectively.

**Additional file 1: Table S4.** Top twenty generalized linear mixed models predicting heartworm infection. Models within ΔAICc < 2 from the best fit model were included in model averaging

| GLMM | Model | Predictors included in each model | *k* | ΔAICc | *w_i_* | *r^2^_m_* | *r^2^_c_* |
| --- | --- | --- | --- | --- | --- | --- | --- |
| Resident and transient coyotes using 95% MCP (*n* = 146) | 1 | age class + developed medium + age class * developed medium | 7 | 0.00 | 0.18 | 0.42 | 0.42 |
|  | 2 | age class + developed medium + mosquito habitat + age class * developed medium | 8 | 0.54 | 0.14 | 0.44 | 0.44 |
|  | 3 | age class + year + developed medium + mosquito habitat + age class * developed medium | 10 | 0.63 | 0.13 | 0.49 | 0.49 |
|  | 4 | age class + year + developed medium + age class * developed medium | 9 | 1.43 | 0.09 | 0.45 | 0.45 |
|  | 5 | age class + developed medium + developed low + age class * developed medium | 8 | 1.55 | 0.08 | 0.41 | 0.41 |
|  | 6 | age class + developed medium + resident status + age class * developed medium | 8 | 2.01 | 0.07 | 0.41 | 0.41 |
|  | 7 | age class + developed medium + developed low + mosquito habitat + age class * developed medium | 9 | 2.43 | 0.05 | 0.43 | 0.43 |
|  | 8 | age class + developed medium + mosquito habitat + social status + age class * developed medium | 9 | 2.7 | 0.05 | 0.43 | 0.43 |
|  | 9 | age class + year + developed medium + developed low + mosquito habitat + age class * developed medium | 11 | 2.93 | 0.04 | 0.48 | 0.48 |
|  | 10 | age class + year + developed medium + mosquito habitat + social status + age class * developed medium | 11 | 2.96 | 0.04 | 0.49 | 0.49 |
|  | 11 | age class + year + developed medium + developed low + age class * developed medium | 10 | 3.47 | 0.03 | 0.44 | 0.44 |
|  | 12 | age class + year + developed medium + social status + age class * developed medium | 10 | 3.71 | 0.03 | 0.44 | 0.44 |
|  | 13 | age class + developed medium + developed low + social status + age class * developed medium | 9 | 3.73 | 0.03 | 0.4 | 0.4 |
|  | 14 | age class + developed medium + developed low + mosquito habitat + social status + age class * developed medium | 10 | 4.68 | 0.02 | 0.42 | 0.42 |
|  | 15 | age class + year + developed medium + developed low + mosquito habitat + social status + age class * developed medium | 12 | 5.29 | 0.01 | 0.49 | 0.49 |
|  | 16 | age class + year + developed medium + social status + age class * medium developed | 11 | 5.8 | 0.01 | 0.43 | 0.43 |
|  | 17 | age class | 4 | 8.71 | 0.002 | 0.13 | 0.17 |
|  | 18 | age class + mosquito habitat | 5 | 9.47 | 0.002 | 0.14 | 0.16 |
|  | 19 | age class + developed medium | 5 | 9.5 | 0.002 | 0.15 | 0.15 |
|  | 20 | age class + social status | 5 | 9.86 | 0.001 | 0.14 | 0.17 |
| Resident coyotes only using 95% MCP (*n* = 107) | 1 | age class + developed medium + age class * developed medium | 7 | 0.00 | 0.23 | 0.63 | 0.63 |
|  | 2 | age class + developed medium + mosquito habitat + age class * developed medium | 8 | 0.99 | 0.14 | 0.68 | 0.68 |
|  | 3 | age class | 4 | 1.57 | 0.1 | 0.12 | 0.16 |
|  | 4 | age class + developed medium | 5 | 2.13 | 0.08 | 0.15 | 0.15 |
|  | 5 | age class + developed medium + developed low + age class * developed medium | 8 | 2.21 | 0.08 | 0.63 | 0.63 |
|  | 6 | age class + mosquito habitat | 5 | 2.58 | 0.06 | 0.14 | 0.14 |
|  | 7 | age class + developed medium + developed low + mosquito habitat + age class * developed medium | 9 | 3.33 | 0.04 | 0.67 | 0.67 |
|  | 8 | age class + developed low | 5 | 3.74 | 0.04 | 0.12 | 0.15 |
|  | 9 | age class + developed medium + mosquito habitat | 6 | 3.78 | 0.04 | 0.15 | 0.15 |
|  | 10 | age class + year + developed medium + age class * developed medium | 9 | 3.82 | 0.03 | 0.67 | 0.67 |
|  | 11 | age class + year + developed medium + mosquito habitat + age class * + developed medium | 10 | 3.98 | 0.03 | 0.73 | 0.73 |
|  | 12 | age class + developed medium + developed low | 6 | 4.34 | 0.03 | 0.15 | 0.15 |
|  | 13 | age class + developed low + mosquito habitat | 6 | 4.79 | 0.02 | 0.14 | 0.14 |
|  | 14 | age class + year | 6 | 5.05 | 0.02 | 0.12 | 0.18 |
|  | 15 | age class + year + mosquito habitat | 7 | 5.12 | 0.02 | 0.16 | 0.16 |
|  | 16 | age class + year + developed medium | 7 | 5.27 | 0.02 | 0.16 | 0.16 |
|  | 17 | intercept | 2 | 5.94 | 0.01 | 0 | 0.07 |
|  | 18 | age class + developed medium + developed low + mosquito habitat | 7 | 5.99 | 0.01 | 0.15 | 0.15 |
|  | 19 | age class + year + developed medium + developed low + age class * + developed medium | 10 | 6.18 | 0.01 | 0.66 | 0.66 |
|  | 20 | age class + year + developed medium + mosquito habitat | 8 | 6.25 | 0.01 | 0.18 | 0.18 |
| Resident and transient coyotes using 95%  LoCoH-a (*n* = 146) | 1 | age class + + developed medium + age class * developed medium | 7 | 0.00 | 0.16 | 0.45 | 0.45 |
|  | 2 | age class + developed medium + mosquito habitat + age class * developed medium | 8 | 0.22 | 0.15 | 0.46 | 0.46 |
|  | 3 | age class + year + developed medium + mosquito habitat + age class * developed medium | 10 | 0.48 | 0.13 | 0.5 | 0.5 |
|  | 4 | age class + developed medium + developed low + age class * developed medium | 8 | 1.26 | 0.09 | 0.44 | 0.44 |
|  | 5 | age class + year + developed medium + age class * developed medium | 9 | 1.31 | 0.08 | 0.48 | 0.48 |
|  | 6 | age class + social status + developed medium + age class * developed medium | 8 | 1.94 | 0.06 |  |  |
|  | 7 | age class + developed medium + developed low + mosquito habitat + age class * developed medium | 9 | 1.95 | 0.06 | 0.45 | 0.45 |
|  | 8 | age class + social status + developed medium + mosquito habitat + age class * developed medium | 9 | 2.27 | 0.05 |  |  |
|  | 9 | age class + year + developed medium + developed low + mosquito habitat + age class * developed medium | 11 | 2.72 | 0.04 | 0.5 | 0.5 |
|  | 10 | age class + year + social status + developed medium + mosquito habitat + age class * developed medium | 11 | 2.82 | 0.04 |  |  |
|  | 11 | age class + year + developed medium + developed low + age class * developed medium | 10 | 3.14 | 0.03 | 0.47 | 0.47 |
|  | 12 | age class + social status + developed medium + developed low + age class * developed medium | 9 | 3.28 | 0.03 |  |  |
|  | 13 | age class + year + social status + developed medium + + age class * developed medium | 10 | 3.56 | 0.03 |  |  |
|  | 14 | age class + social status + developed medium + developed low + mosquito habitat + age class * developed medium | 10 | 4.06 | 0.02 |  |  |
|  | 15 | age class + year + social status + developed medium + developed low + mosquito habitat + age class * developed medium | 12 | 5.09 | 0.01 |  |  |
|  | 16 | age class + year + social status + developed medium + developed low + age class * developed medium | 11 | 5.43 | 0.01 |  |  |
|  | 17 | age class | 4 | 13.77 | 0.00 |  |  |
|  | 18 | age class + mosquito habitat | 5 | 14.31 | 0.00 |  |  |
|  | 19 | age class + social status | 5 | 14.92 | 0.00 |  |  |
|  | 20 | age class + developed medium | 5 | 15.01 | 0.00 |  |  |
| Resident coyotes only using 95% LoCoH-a (*n* = 107) | 1 | age class + developed medium + age class * developed medium | 7 | 0.00 | 0.28 | 0.45 | 0.45 |
|  | 2 | age class + developed medium + mosquito-habitat + age class * developed medium | 8 | 0.78 | 0.19 | 0.47 | 0.47 |
|  | 3 | age class + developed medium + developed low + age class * developed medium | 8 | 1.96 | 0.11 | 0.44 | 0.44 |
|  | 4 | age class | 4 | 2.66 | 0.08 | 0.12 | 0.16 |
|  | 5 | age class + developed medium + developed low + mosquito habitat + age class * developed medium | 9 | 2.98 | 0.06 | 0.46 | 0.46 |
|  | 6 | age class + mosquito habitat | 5 | 3.58 | 0.05 | 0.14 | 0.14 |
|  | 7 | age class + year + developed medium + age class * developed medium | 9 | 4.01 | 0.04 | 0.47 | 0.47 |
|  | 8 | age class + year + developed medium + mosquito habitat + age class * developed medium | 10 | 4.38 | 0.03 | 0.51 | 0.51 |
|  | 9 | age class + developed medium | 5 | 4.54 | 0.03 | 0.13 | 0.13 |
|  | 10 | age class + developed low | 5 | 4.85 | 0.03 | 0.12 | 0.16 |
|  | 11 | age class + developed medium + mosquito-habitat | 6 | 5.63 | 0.02 | 0.14 | 0.14 |
|  | 12 | age class + developed low + mosquito-habitat | 6 | 5.80 | 0.02 | 0.14 | 0.14 |
|  | 13 | age class + year | 6 | 6.15 | 0.01 | 0.12 | 0.18 |
|  | 14 | age class + year + developed medium + developed low + age class * developed medium | 10 | 6.15 | 0.01 | 0.46 | 0.46 |
|  | 15 | age class + year + mosquito habitat | 7 | 6.5 | 0.01 | 0.16 | 0.16 |
|  | 16 | age class + developed medium + developed low | 6 | 6.77 | 0.01 | 0.13 | 0.13 |
|  | 17 | age class + year + developed medium + developed low + mosquito habitat + age class * developed medium | 11 | 6.8 | 0.009 | 0.5 | 0.5 |
|  | 18 | mosquito habitat | 3 | 6.98 | 0.009 | 0.04 | 0.04 |
|  | 19 | intercept | 2 | 7.03 | 0.008 | 0 | 0.07 |
|  | 20 | age class + year + developed medium | 7 | 7.9 | 0.005 | 0.14 | 0.14 |

*k* represents the number of estimated coefficients in each model, *ΔAICc* the difference in AICc between a given model and the top model, *w_i_* the renormalized Akaike weights, *r*^2^ the coefficient of determination.

**Additional file 1: Table S5.** Model averaging results from binomial generalized linear mixed models of the probability of heartworm infection in coyotes (*n* = 146) using the adaptive local convex hull (a-LoCoH). Predictors were obtained from the top ranking models (ΔAICc < 2; Table S4). Significant terms are depicted in bold

| **Model** | **Predictor** | **Estimate** | **SE** | **z** | **Pr(>\|z\|)** | **Mean OR** | **95% CI** |
| --- | --- | --- | --- | --- | --- | --- | --- |
| Resident and transient coyotes  (*n* = 146) | (intercept) | -0.69 | 0.43 | 1.62 | 0.11 | 0.5 | (0.22 – 1.16) |
|  | age class (subadult) | -0.67 | 0.48 | 1.41 | 0.16 | 0.51 | (0.2 – 1.3) |
|  | age class (pup) | -2.23 | 0.99 | 2.26 | 0.02 | 0.11 | (0.02 – 0.75) |
|  | social status (transient) | -0.29 | 0.55 | 0.54 | 0.59 | 0.74 | (0.25 – 2.19) |
|  | proportion low developed land in home range | -0.22 | 0.26 | 0.86 | 0.39 | 0.8 | (0.48 – 1.33) |
|  | proportion medium developed land in home range | -0.78 | 0.39 | 1.99 | 0.05 | 0.46 | (0.21 – 0.99) |
|  | proportion mosquito habitat in home range | 0.32 | 0.23 | 1.41 | 0.16 | 1.38 | (0.88 – 2.17) |
|  | year | -1.56 | 1.57 | 0.99 | 0.32 | 0.21 | (0.01 – 4.57) |
|  | year (quadratic) | 0.82 | 0.66 | 1.26 | 0.21 | 2.28 | (0.63 – 8.22) |
|  | age class (subadult) * proportion medium developed | 1.98 | 0.57 | 3.45 | 0.0006 | 7.21 | (2.35 – 22.17) |
|  | age class (pup) ***** proportion medium developed | -1.17 | 1.37 | 0.86 | 0.39 | 0.31 | (0.02 – 4.52) |
| Residents only (*n* = 107) | (intercept) | -0.51 | 0.29 | 1.76 | 0.08 | 0.6 | (0.34 – 1.06) |
|  | age class (subadult) | -0.73 | 0.51 | 1.43 | 0.15 | 0.48 | (0.18 – 1.31) |
|  | age class (pup) | -2.67 | 1.76 | 1.52 | 0.13 | 0.07 | (0.00 – 2.19) |
|  | proportion developed medium land in home range | -0.55 | 0.36 | 1.53 | 0.13 | 0.58 | (0.29 – 1.16) |
|  | proportion developed low land in home range | -0.17 | 0.28 | 0.6 | 0.55 | 0.85 | (0.49 – 1.46) |
|  | proportion mosquito habitat in home range | 0.29 | 0.25 | 1.18 | 0.24 | 1.34 | (0.83 – 2.18) |
|  | age class (subadult) * proportion medium developed | 1.45 | 0.58 | 2.5 | 0.01 | 4.28 | (1.37 – 13.41) |
|  | age class (pup) * proportion medium developed | -1.55 | 2.51 | 0.62 | 0.54 | 0.21 | (0.00 – 29.11) |

Significant terms are depicted in bold (with 95% confidence intervals (CI) not overlapping with 1 and *P* < 0.05). *SE* is the standard error, Pr(>|z|) the *P*-value associated with the *z* statistic, and mean *OR* the mean odds ratio.
